# An Open Trial for Transdiagnostic Behavior Therapy for Primary Care (TBT-PC) in Veterans with Symptoms of Depression and Anxiety

**DOI:** 10.3390/bs15091287

**Published:** 2025-09-20

**Authors:** Daniel F. Gros, Stephanie Hart, Michelle Pompei, Ron Acierno

**Affiliations:** 1 Mental Health Service 116, Ralph H. Johnson VA Healthcare System, 109 Bee Street, Charleston, SC 29401, USA; zeigls@musc.edu (S.H.); pompei@musc.edu (M.P.); ronald.acierno@uth.tmc.edu (R.A.); 2 Department of Psychology & Behavioral Sciences, Medical University of South Carolina, Charleston, SC 29425, USA; 3 Louis A. Faillace, MD, Department of Psychiatry and Behavioral Sciences, University of Texas Health Science Center at Houston, Houston, TX 77054, USA

**Keywords:** transdiagnostic, depression, anxiety, veterans

## Abstract

Transdiagnostic psychotherapies were developed to address multiple diagnostic presentations via a single, easier-to-implement protocol. However, despite advances, these protocols are largely limited to longer formats designed for mental health settings. The present study investigated a version of Transdiagnostic Behavior Therapy (TBT-PC) that was adapted for primary care settings. Forty-one participants with emotional disorders received 6 sessions of TBT-PC, with symptoms of depression and anxiety assessed at baseline, immediate post-treatment, and at 3-month follow-up. Medium treatment effects for symptoms of depression and anxiety were evidenced at post-treatment and sustained at follow-up. Treatment feasibility was supported by excellent attendance, treatment completion, and patient satisfaction scores. Together, the present findings provide initial support for TBT-PC in primary care patients with emotional disorders.

## 1. Introduction

Emotional disorders are a set of psychiatric diagnoses characterized by strong affective disturbance (Nesse & Ellsworth, 2009; Watson & Naragon-Gainey, 2014). Example emotional disorders include major depressive disorder, panic disorder, generalized anxiety disorder, and posttraumatic stress disorder (PTSD). These conditions are associated with occupational, educational, and social impairments (Hohls et al., 2021), with high societal costs (Chisholm et al., 2016). However, despite mental health-focused symptom severity and functional impairment, individuals with emotional disorders are more likely to seek care within primary care settings than in specialized mental health treatment facilities (Coyne et al., 2002). Unfortunately, many patients with emotional disorders are not adequately assessed, diagnosed, and treated in these primary care settings (Wittchen et al., 2003).

Over the years, many efforts have focused on incorporating mental health services into primary care settings to better address mental health conditions, such as emotional disorders (Zeiss & Karlin, 2008). For example, evidence-based psychotherapy protocols, which are some of the most effective treatments for emotional disorders, have been revised into briefer formats to better fit within the primary care model (i.e., no more than six 30 min sessions) (Funderburk et al., 2025). These protocols cover a wide range of presenting problems, including specific symptoms (e.g., Brief Cognitive Behavioral Therapy for Insomnia), generalized symptoms across conditions (e.g., Problem-Solving Therapy), or specific emotional disorders (e.g., Brief Behavioral Activation for Depression), and have been used successfully to address the symptoms of emotional disorders for many years (Fordham et al., 2021; Funderburk et al., 2021; Lorenzo-Luaces et al., 2021). However, despite their efficacy across conditions, implementation of these protocols has been limited in part due to the vast number of protocols required to learn to treat each of the emotional disorders (Barlow et al., 2020; Gros et al., 2016; Hofmann, 2022). More specifically, significant training and financial considerations are required to learn to apply each protocol (e.g., 6 months per protocol per disorder; Karlin & Cross, 2014). Thus, it could take years to learn a sufficient number of protocols to treat each of the emotional disorders, leaving many mental health treatment facilities to struggle to reliably offer high-quality psychotherapies to their patients across diagnoses (Ecker et al., 2022).

Many solutions have been proposed to improve implementation of psychotherapies across settings with mixed success (McHugh et al., 2023). One promising solution has been to shift from a disorder-specific to a transdiagnostic approach to the evidence-based psychotherapis for emotional disorders (Barlow et al., 2020; Gros et al., 2016). Transdiagnostic practices are defined as being “equipped to address psychopathology across diagnostic boundaries, allowing them to parsimoniously target comorbid conditions and reduce therapist training burden.” (Sauer-Zavala et al., 2017, p. 135). More specifically, one transdiagnostic psychotherapy protocol could be used to treat multiple disorders compared to learning separate disorder-specific protocols to treat each disorder separately. In terms of traditional, face-to-face psychotherapy practices, several transdiagnostic protocols have been developed and investigated in the literature, including Group Transdiagnostic Cognitive-Behavioral Therapy (Norton, 2012), Transdiagnostic Behavior Therapy (TBT) (Gros, 2014), and Unified Protocol for Transdiagnostic Treatment of Emotional Disorders (UP) (Barlow et al., 2017). Across protocols, transdiagnostic treatments demonstrate large treatment effects, superiority to nontherapeutic control conditions, and comparable outcomes to select disorder-specific psychotherapies across patients with a wide range of symptom presentations, impairments, and diagnoses (Cuijpers et al., 2023; Schaeuffele et al., 2024).

To date, investigation of transdiagnostic psychotherapies in primary care has been limited largely to group psychotherapies (Vogel et al., 2024). And while most of these transdiagnostic group psychotherapies show promising results (Cano-Vindel et al., 2022; Ejeby et al., 2014), they generally are too time-consuming to fit within traditional primary care settings (e.g., 12 weekly 90 min sessions) (Funderburk et al., 2025). Briefer transdiagnostic protocols include a five-session 90 min group version of the UP that removed modules deemed less critical for primary care patients (De Paul & Caver, 2021) and a five-session 120 min group cognitive behavioral therapy focused on challenging negative thought patterns (Kristjánsdóttir et al., 2016). Although pilot trials of both interventions demonstrated significant symptom improvements, the duration of the therapy sessions (90–120 min) is inconsistent with primary care treatment guidelines and may limit their implementation as a result (Funderburk et al., 2025).

Given the limitations of transdiagnostic psychotherapies in primary care settings to date, additional research and development of the available protocols are needed to improve options for future implementation. One possible candidate for further consideration is TBT. Unlike alternative protocols that rely on multiple treatment modules within a single protocol (e.g., 8 modules delivered over 12–18 sessions; Barlow et al., 2017), TBT involves a singular behavioral treatment component (i.e., exposure) to address transdiagnostic avoidance across the emotional disorders (Gros, 2014). Studies of TBT show the treatment effectively reduces impairment across multiple domains of functioning in patients presenting with a wide range of diagnoses and comorbidities, and with similar effects to established disorder-specific psychotherapies (Gros & Allan, 2019). Although traditionally delivered over 8–12 weekly 60 min sessions, a primary care version of the TBT protocol may be possible by reducing session content without removing the primary/only treatment component.

The present study investigated a shortened version of TBT designed for a primary care setting (TBT-PC) in primary care patients with emotional disorders. The revision of TBT into TBT-PC was informed by previous studies of and clinical experiences with TBT, led by the original author of the TBT protocol (DG), and involved: (1) reducing psychoeducation on the emotional disorders in Sessions 1–2 into a single session, (2) reducing the number of examples to explain the transdiagnostic symptom target (avoidance) and transdiagnostic approach to treatment (exposure) in Sessions 3–4 into a single session, (3) reducing the number of sessions to practice and problem-solve exposure practices from 7 to 3, and (4) removing the optional modules for associated symptoms (e.g., sleep or anger). In general, all session content was reduced to target 30 min session duration. This initial open trial was designed to assess the treatment effects (e.g., self-reported symptom outcomes) and basic feasibility (e.g., treatment completion and patient satisfaction) of TBT-PC in veterans with emotional disorders in preparation for future randomized clinical trials. Of note, TBT is one of the only transdiagnostic psychotherapies to be thoroughly studied in veteran samples (Schaeuffele et al., 2024). Based on findings from similar studies of individual disorder-specific psychotherapies in primary care settings (Funderburk et al., 2021), it was hypothesized that patients receiving TBT-PC would demonstrate reductions in symptoms of depression and anxiety, with medium treatment effects. In addition, TBT-PC would be well-received by patients as demonstrated by high rates of treatment completion as well as patient satisfaction scores.

## 2. Materials and Methods

### 2.1. Participants

Forty-one treatment-seeking patients with symptoms of depression and anxiety were recruited from primary care within a large Southeastern Veteran Affairs Healthcare System (VAHCS) and neighboring active duty military sites (e.g., Army base and Naval hospital). Eligible participants were required to be: (1) active-duty personnel or veterans of the United States armed services, (2) aged 18 years or above, (3) able to provide informed consent, and (4) scored 10 or above at baseline on the PHQ-9 to support clinically significant symptoms and potentially benefit for psychotherapy (Kroenke et al., 2001). Patients with active symptoms of a psychotic disorder or severely impaired memory (e.g., dementia), active suicidal ideation with clear intent, or severe substance use disorders were excluded.

### 2.2. Study Procedures

All procedures were approved by the local VAHCS Research and Development committee as well as the Institutional Review Board at the affiliated university. Participants were recruited from October 2024 to May 2025. Agreeable, treatment-seeking patients were scheduled for an intake appointment to complete consent documentation, a diagnostic interview, and self-report questionnaires. The intake assessment was completed by a master’s-level clinician with years of experience administering diagnostic assessments, such as the Mini International Neuropsychiatric Interview 7.0 (MINI-7). Upon completion of the intake and confirmation that inclusion/exclusion criteria were met, participants were assigned to complete 6 weekly sessions of TBT-PC. TBT-PC was delivered by master’s-level therapists with years of experience delivering evidence-based psychotherapies to veterans and service members. All therapists completed a 4 h training on TBT-PC (Gros et al., 2017) and received ongoing supervision from the lead author of TBT-PC during the course of the trial (DG). Participants repeated the self-report measures at immediate post-treatment (one week after completion of session 6) and at follow-up (three months after completion of session 6). Participants were compensated up to $100 USD for their completion of the study measures. All questionnaires were administered via REDCap, a secure web application for building and managing online surveys and databases.

### 2.3. Transdiagnostic Behavior Therapy for Primary Care

The original, full protocol for TBT was designed to address overall psychiatric well-being in patients via reengagement in significant activities, relationships, and community involvements that are typically avoided due to psychiatric symptomatology (Gros, 2014; Gros & Allan, 2019). Avoidance is the transdiagnostic symptom targeted in TBT (Gros, 2014; Gros et al., 2023). More specifically, the protocol seeks to challenge four different types of avoidance associated with negative and positive emotions (situational, physical, thought, and positive emotional avoidance). Situational, interoceptive, imaginal, and positive emotional exposure practices are used to reduce avoidance across the four types and lead to decreases in negative emotions and increases in positive emotions as well as improvements in overall well-being and social functioning (Gros, 2014). As noted earlier, the original protocol was revised to create a briefer, targeted protocol for a less symptomatic primary care population that included the following session topics: Session 1: psychoeducation on avoidance, Session 2: using exposures to overcome avoidance, Session 3: learning the details of exposure therapy, Session 4: exposure implementation, Session 5: maintenance of exposure practices, and Session 6: relapse prevention. Daily exposure practices were assigned as homework as of Session 3.

### 2.4. Measures

#### Charleston Psychiatric Outpatient Satisfaction Scale (CPOSS)

The CPOSS is 16-item measure designed to assess patient satisfaction related to key clinical (e.g., helpfulness of the services you have received), administrative (e.g., helpfulness of the secretary), and environmental (e.g., location of outpatient service) factors in psychiatric outpatient settings (Frueh et al., 2002). All items are assessed on a 1–5 scale (“poor” to “excellent”). The CPOSS has been shown to contain separate scales for respectful care, appearance of facility, convenience of facility, and recommendation to a friend or family member, as well as demonstrating good reliability and validity (Gros et al., 2013). Due to the design of the present study (e.g., primarily delivered via telehealth), only the respectful care scale (CPOSS-RC) was investigated. The CPOSS-RC contains eight items scored on a 1–5 scale, with scale range of 8 to 40. The CPOSS-RC demonstrated good internal consistency at immediate post-treatment (α = 0.93).

### 2.5. Generalized Anxiety Disorder-7 (GAD-7)

The GAD-7 is a 7-item self-reported scale designed to assess the symptoms and diagnosis of generalized anxiety disorder “over the last 2 weeks”, with items scored on 0–3 scale (“Not at all” to “Nearly every day”) and with a scale range of 0 to 21 (Spitzer et al., 2006). The GAD-7 has been shown to have good reliability as well as validity (Spitzer et al., 2006). The GAD-7 demonstrated good internal consistency across all assessment points (αs ≥ 0.77).

### 2.6. Mini International Neuropsychiatric Interview 7.0 (MINI-7)

The MINI 7.0 is one of the most used standardized structured interviews for the 17 most common psychiatric diagnoses according to the DSM-5 (Sheehan et al., 1997, 1998). Although the psychometric properties of the MINI were not re-evaluated in the present study, the MINI has demonstrated similar findings for sensitivity, specificity, and inter-rater reliability to more lengthy diagnostic interviews in previous research (Sheehan et al., 1998). The MINI was used to assess diagnostic status at baseline to better describe the characteristics of the treatment-seeking sample. Given the short duration of the treatment and time-frames for diagnostic criteria (e.g., absence of symptoms for two months to be considered full remission), change in diagnostic status was not anticipated and was not evaluated in the present study.

### 2.7. PHQ-9

The PHQ-9 is a 9-item self-reported depression scale derived from the Patient Health Questionnaire to assess the symptoms and diagnosis of depression “over the last 2 weeks”, with items scored on 0–3 scale (“Not at all” to “Nearly every day”) and with a scale range of 0 to 27 (Kroenke et al., 2001). The PHQ-9 has been shown to have good reliability as well as validity (Kroenke et al., 2001). The PHQ9 demonstrated good internal consistency across all assessment points (αs ≥ 0.71).

### 2.8. Data Analysis

All analyses were conducted using SPSS Version 29 (IBM Corporation, Armonk, NY, USA). An inspection of all data was completed prior to analyses. Although no missing data was observed in the baseline assessment, missing data was present at post-treatment (*n* = 2) and three-month follow-up (*n* = 17) due to treatment discontinuation and failure to follow up. Separate analysis of variance tests revealed no differences in completers and non-completers in baseline (post-treatment and three-month follow-up) and post-treatment (three-month follow-up) on the PHQ-9 and GAD-7 scales across timepoints (*p*s > 0.31). Descriptives (mean, standard deviation) were run for PHQ-9 and GAD-7 at baseline, as well as the CPOSS-RC at post-treatment. Paired-samples t-tests were used to investigate changes from baseline to post-treatment and baseline to three-month follow-up. Cohen’s *d* (Cohen, 1988) and the reliable change index (Jacobson & Truax, 1992) were calculated to estimate the size and reliability of the treatment effects.

## 3. Results

### 3.1. Demographics and Baseline Symptoms

A full description of the demographics of the sample was presented in Table 1. In brief, the average participant was 40.3 years old (*SD* = 13.7), male (*n* = 34; 82.9%), White (*n* = 17; 41.5%; Black or African American: *n* = 17; 41.5%), and served in the Operations Enduring Freedom, Iraqi Freedom, or New Dawn (*n* = 20; 48.8%). Most participants were diagnosed with either major depressive disorder (*n* = 38; 92.7%) or PTSD (*n* = 29; 70.7%) on the MINI 7.0. Participants endorsed moderate-to-severe symptoms of depression on the PHQ-9 (*M* = 17.3; *SD* = 4.8; range of 10 to 25) and mild-to-severe symptoms of anxiety on the GAD-7 (*M* = 14.4; *SD* = 4.6; range of 6 to 21) at baseline.

### 3.2. Treatment Outcome and Feasibility

Of the full treatment sample, 39 of 41 (95.1%) completed TBT-PC, with 92.7% (*n* = 38) completing all six sessions of the protocol (one participant completed only 5 sessions, but still completed the protocol). However, only half of participants completed the three-month follow-up assessment (24 of 39; 53.9%). Treatment outcome findings are presented in Table 2. Participants demonstrated significant reductions in the symptoms of depression with a medium effect (*p* = 0.001; *d* = 0.53) and significant reductions in the symptoms of anxiety with a small-to-medium effect (*p* = 0.017; *d* = 0.29). These treatment effects remained significant and with consistent effect sizes at three-month follow-up (*p*s < 0.036; *d*s > 0.43). The reliable change index of 3.1 for the PHQ-9 and 2.5 for the GAD-7 were based on normative psychometric data from treatment-seeking veterans (Ahmadi et al., 2023; Jacobson & Truax, 1992). Based on these calculations, participants did not evidence reliable change at immediate post-treatment on the PHQ-9 (2.6) or GAD-7 (1.3) but did on the PHQ-9 (3.7) at the three-month follow-up assessment. The GAD-7 score improved at three-month follow-up but remained slightly below reliable change index (2.2). Participants also endorsed high satisfaction with TBT-PC on the CPOSS-RC (*M* = 25.9; *SD* = 5.3).

## 4. Discussion

The present study investigated a version of TBT designed to address the symptoms of emotional disorders in a primary care setting. In contrast to the full TBT protocol, TBT-PC was designed to match recommended format for primary care in terms of fewer sessions (6) and a shorter session duration (30 min) (Funderburk et al., 2025). Findings supported medium treatment effects for TBT-PC in primary care patients with emotional disorders, including major depressive disorder and PTSD. These findings were also consistent with reliable symptom changes at the three-month follow-up assessment for the symptoms of depression (Jacobson & Truax, 1992). Participants evidenced excellent attendance and high satisfaction in support of the feasibility of TBT-PC. Together, the present findings provide initial support for TBT-PC in primary care patients with emotional disorders.

Although lacking a comparison group, the initial findings for TBT-PC were promising. The immediate post-treatment effect sizes for TBT-PC were generally smaller than findings for the full TBT protocol in mental health settings (Gros & Allan, 2019), but roughly consistent with brief disorder-specific psychotherapies for depression and mixed depression-anxiety in the literature (Cape et al., 2010). Consistent with the goals of mental health services in primary care (Zeiss & Karlin, 2008), initial symptom improvements, even if only modest, could lead to improved perceptions in primary care patients with emotional disorders and lead to improved engagement in mental health services in the future (Wray et al., 2019). In addition, the transdiagnostic treatment perspective of TBT-PC may be easier to implement in a diverse patient setting in primary care compared to traditional disorder-specific approaches (Gros et al., 2017).

If further supported in future investigations, there may be important clinical implications for TBT-PC. Given the application to primary care settings and transdiagnostic treatment perspective, TBT-PC could be delivered to the majority of patients with emotional disorders presenting in primary care settings. To date, the full version of TBT has been supported in patients with trauma and stressor-related disorders (PTSD), depressive disorders (major depressive disorder), and anxiety disorders (panic disorder, agoraphobia, social anxiety disorder, generalized anxiety disorder) (Gros, 2019). TBT-PC is expected to demonstrate similar effects across disorders as it includes the same primary treatment component, although abbreviated. In addition, TBT has been shown to be easily disseminated and implemented across providers and settings in an initial investigation (Gros et al., 2017), suggesting that an application to a primary care setting and with primary care providers is feasible. Together, if further support is provided for its efficacy via well-designed clinical trials, TBT-PC may be found to be an important tool for addressing the symptoms of emotional disorders in primary care.

The present study involved several limitations that should be addressed in future, larger studies of TBT-PC. The present sample was small and did not include a comparison group, limiting the interpretability of the symptom treatment effects. The study also did not assess for diagnostic changes post-treatment to determine the clinical significance of the symptom change (e.g., did symptom improvements lead to diagnostic remission), nor were self-report measures of PTSD included. Neither the reliability of the diagnostic assessment (inter-rater reliability) nor the fidelity of the delivery of TBT-PC were systematically assessed in the present study, relying instead on supervision within the project team.

Together, the present study provided preliminary support for a transdiagnostic psychotherapy for primary care patients with emotional disorders. In contrast to previous treatments that either relied on longer formats or group presentations (Vogel et al., 2024), TBT-PC may represent an easy-to-implement individual protocol that may be effective across diagnostic presentations. Future research on TBT-PC should involve larger samples and a comparison group to more fully understand the potential benefits of the treatment in a primary care setting.

## Figures and Tables

**Table 1:** Baseline Demographic and Military Characteristics.

Characteristic		
Age	40.3	13.7
Race/Ethnicity		
White	17	41.5%
Black	17	41.5%
Asian	1	2.4%
Latino	5	12.2%
Native American	1	2.4%
Sex		
Male	34	82.9%
Female	7	17.1%
Relationship		
Never Married	12	29.3%
Married	17	41.5%
Separated/Divorced	11	26.8%
Widowed	1	2.4%
Employment		
Unemployed	18	43.9%
Employed	23	56.1%
Education		
High School Graduate	9	22.0%
Some College	19	46.4%
Bachelors	7	17.1%
Graduate School	6	14.6%
Disability ^a^		
Disabled	32	78.0%
Service Branch		
Air Force	3	7.3%
Army	23	56.1%
Coast Guard	2	4.9%
Marine Corps	9	22.0%
Navy	4	9.8%
Theater		
Desert Storm/Shield	8	19.5%
OEF/OIF	20	48.8%
Other	2	4.9%
None	11	26.8%
Combat		
Yes	22	53.7%

Note. ^a^ Disability percentage is calculated for those who indicated they were service-connected.

**Table 2:** Treatment effects for TBT-PC.

Scale	Pre-Tx (39)	Post-Tx (39)	3-Month (24)	Post-Tx *t*	Post-Tx *d*	3-Month *t*	3-Month *d*
PHQ-9	17.3 (4.8)	14.7 (5.0)	13.9 (5.6)	3.5 **	0.53	2.9 *	0.68
GAD-7	14.5 (4.6)	13.2 (4.8)	11.8 (5.4)	2.5 *	0.28	2.2 *	0.44

Note. Ns are presented in parentheses next to column headings. Pre-Tx, Post-Tx, and 3-Month follow-up columns are presented as means (standard deviations). Tx = treatment. ** *p* < 0.01; * *p* < 0.05.

## Data Availability

Data available upon request of the principal investigator.
